# Does early and aggressive management of significant extrusion of the femoral head affect the outcome of Perthes’ disease with the age of onset younger than 7 years?

**DOI:** 10.1007/s12306-021-00709-8

**Published:** 2021-04-18

**Authors:** K. A. Singh, N. Harne, H. Shah

**Affiliations:** Department of Paediatric Orthopaedics, Kasturba Hospital, Kasturba Medical College, Manipal Academy of Higher Education, Manipal, Karnataka 576104 India

**Keywords:** Perthes disease, Age of onset less than 7 years, Containment, Extrusion, SDS

## Abstract

**Background:**

Little literature exists regarding aggressive treatment of the extrusion in the early stage of the disease and the outcome at skeletal maturity. The purpose of the study was to evaluate the outcome of the disease with onset younger than 7 years, treated in the early stage of the disease, with aggressive management of significant extrusion (immediate containment with fixed abduction brace in children less than 5 years and varus derotation osteotomy in older children), and reached skeletal maturity.

**Methods:**

All children with the age of onset younger than 7 years of disease during active Perthes disease were prospectively followed. Children with early stages of the disease (modified Elizabethtown classification) and reached skeletal maturity were included (68 children). The extrusion of the femur head was calculated by Reimer’s migration index on both sides. A migration difference 12 % or above was considered as “significant extrusion”. Children without significant extrusion were treated non-operatively; children with significant extrusion were treated with varus derotation osteotomy. The final radiological outcome was assessed by the Stulberg classification and sphericity deviation score (SDS). The independent “*t*” test and Chi-square test were done to compare the difference between the two groups.

**Results:**

The mean age at the onset and the final follow-up was 5.7 years and 15.3 years. The frequency of significant extrusion was 57%. At the final follow-up, an excellent clinical outcome and radiological outcomes (in 88% hips) were noted. There was no significant difference in the Stulberg groups and SDS (sphericity deviation score) in both groups.

**Conclusion:**

The outcome of the children who had the age of onset of the disease less than 7 years was good with early and aggressive management of the extrusion. The reversal of extrusion is associated with a similar result of non-operative children in this age group.

**Level of evidence:**

III.

## Introduction

Although the outcome of Perthes disease, which has an age of onset less than 7 years, is generally good, consistent results are not seen even in this young age group [1]. The extent of involvement and extrusion of the femoral head influence the outcome in these children [2]. The extent of involvement of the femur head cannot be effectively classified in early stage of the disease. Diverse literature exists regarding the frequency of the femur head extrusion in the active stage of disease [3–9]. The significant extrusion during the late stage of fragmentation and the stage of revascularization contributes maximally to the amount of head deformation. The earlier treatment before the advanced stage of fragmentation is associated with a good outcome. Hence, the extrusion of the femoral head is the prime variable that decides the treatment modality in the younger population.

The extent of head involvement is extensively studied [2, 4]. However, very few articles studied the effect of the femur head extrusion in the active stage of the disease. Green et al. showed great influence of the extrusion in the outcome of the disease [3]. The significance of the extrusion has never been objectively defined before. The outcome of children (less than 7 years’ onset) with Perthes disease at skeletal maturity is poorly reported in the literature. The extrusion of the femur head was aggressively managed in the early stage of the disease in our population. The aggressive management of the extrusion is done with immediate containment as soon as presence of significant extrusion (fixed abduction brace in children less than 5 years and varus derotation osteotomy in older children).

The purpose of the study was to evaluate the outcome of the disease with onset younger than 7 years, treated in the early stage of the disease, with aggressive management of extrusion, and reached skeletal maturity.

## Materials and methods

**Fig. 1:**
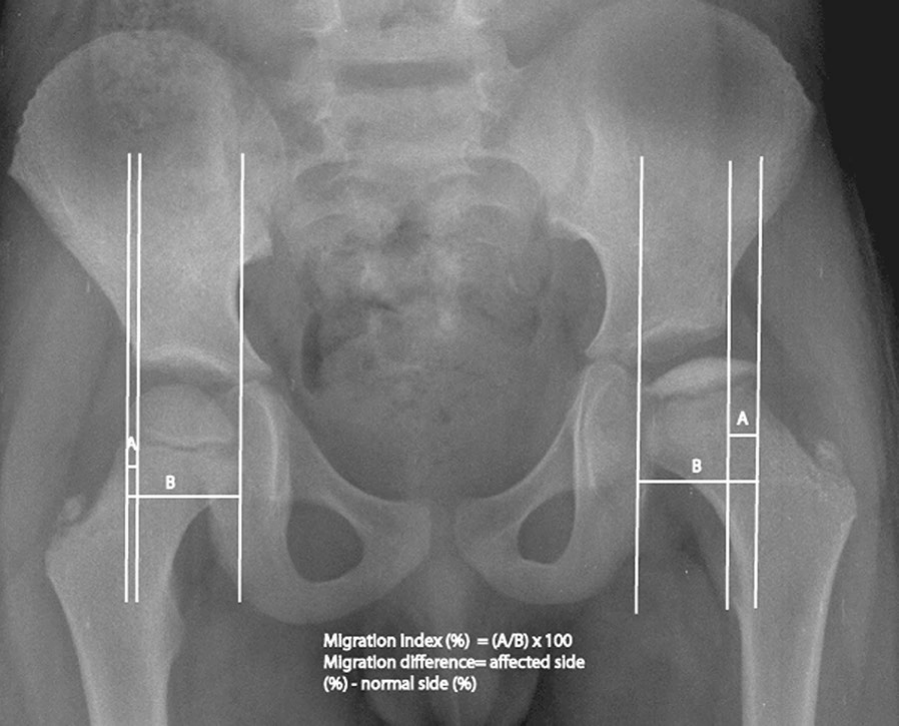
Illustration of Reimer’s migration index on both sides and migration difference calculation

After obtaining Institutional Ethics Committee permission, all consecutive children with Perthes disease were prospectively studied. Over a period of 15 years, 90 children had disease onset before 7 years of age. The modified Elizabethtown classification was used to stage the disease [4]. The early-stage presentation was defined as the presentation in the stage of avascular necrosis or early fragmentation [10]. The extrusion of the femur head was calculated by Reimer’s migration index at the initial presentation and subsequent follow-up [11]. A migration difference 12% or above was considered as “significant extrusion” (Fig. 1) [12]. A significant extrusion was aggressively managed during the early stage of disease (the stage of avascular necrosis and the stage of early fragmentation).

### Treatment protocol

**Fig. 2:**
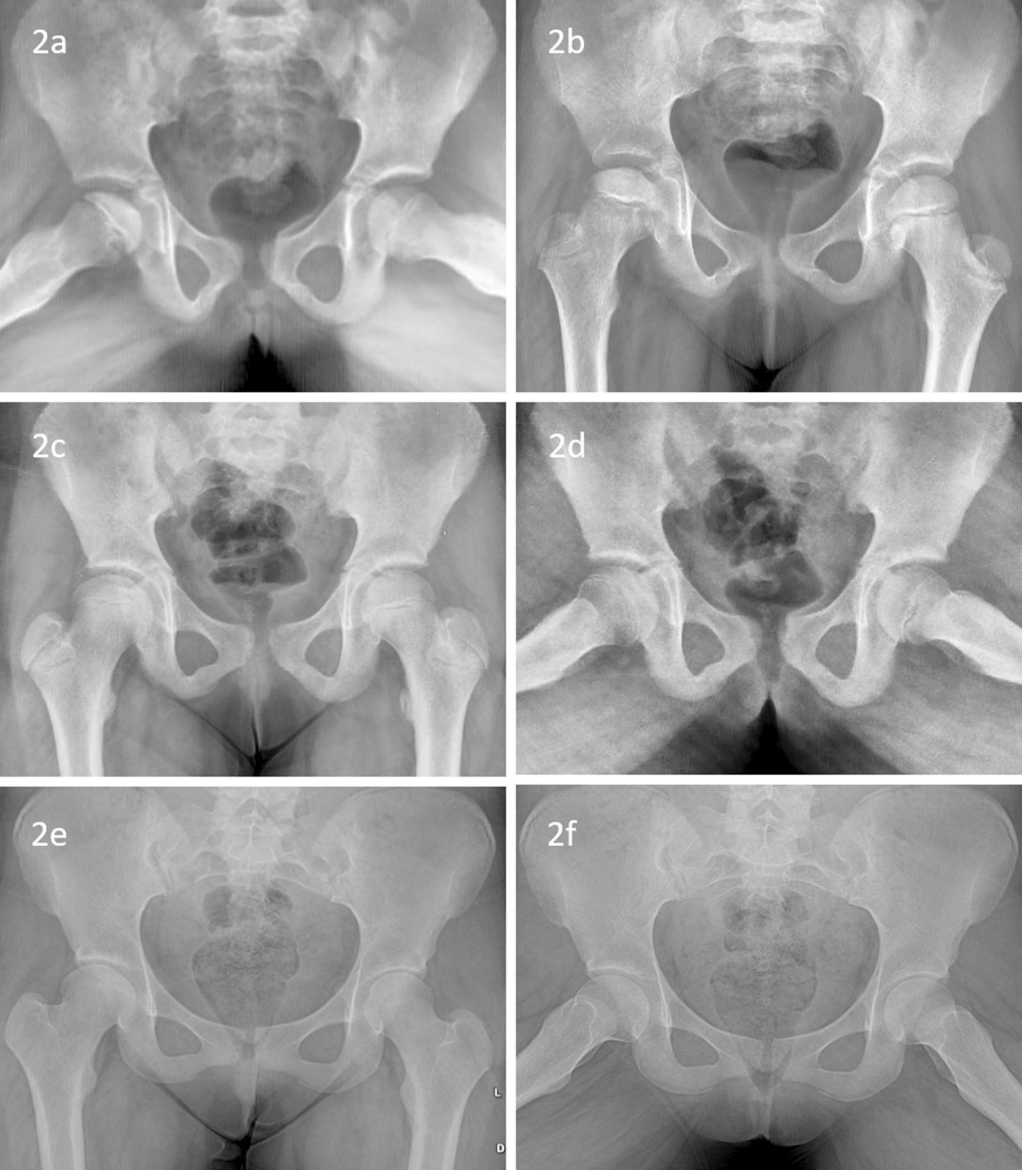
Anteroposterior (**a**) and frog-lateral (**b**) radiographs of a girl with the age of onset 6 years with right side Perthes disease stage Ib without significant extrusion. With non-operative management, the disease healed with spherical and congruent hip (**c**, **d**). At skeletal maturity, spherical congruent hip was noted (**e**, **f**)

All children with initial restriction of the range of motion were treated with skin traction till gaining normal range of movements. Children with severe limitation of the range of motion were treated with broomstick cast for 6 weeks. Children without significant extrusion at the initial presentation were treated non-operatively (Fig. 2). They were kept non-weight bearing with axillary crutches. They were followed up at 3 months’ interval to observe the extrusion. Without significant extrusion till the advanced stage of fragmentation, they were treated non-operatively.

**Fig. 3:**
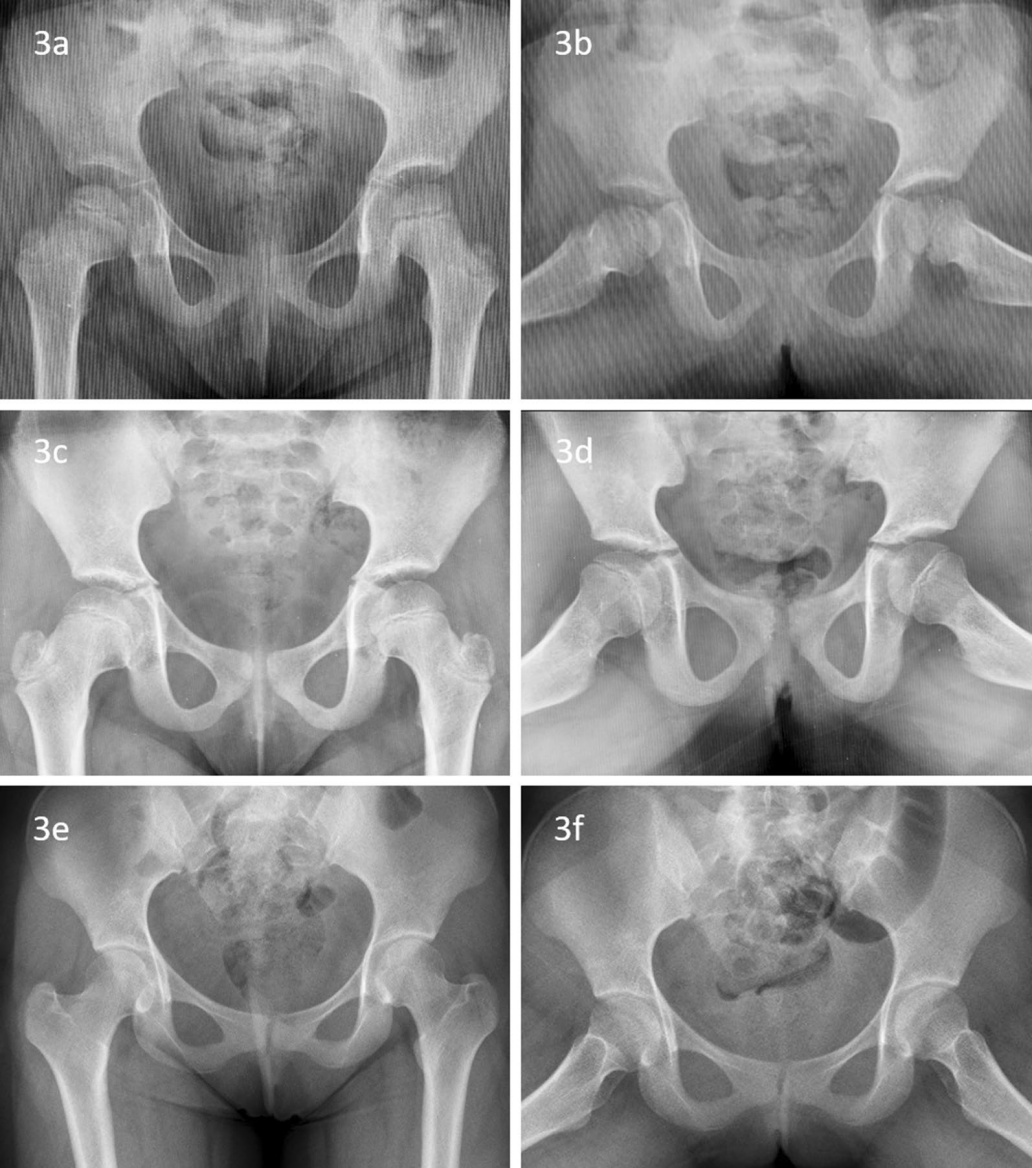
A girl presented with Perthes disease (age of onset 4.5 years) of right hip (**a**, **b**). She was managed with an abduction splint. The femur head was enlarged but spherical and congruent at healed stage (**c**, **d**). At 17 years, the femur head was Stulberg Class I hip (**e**, **f**)

**Fig. 4:**
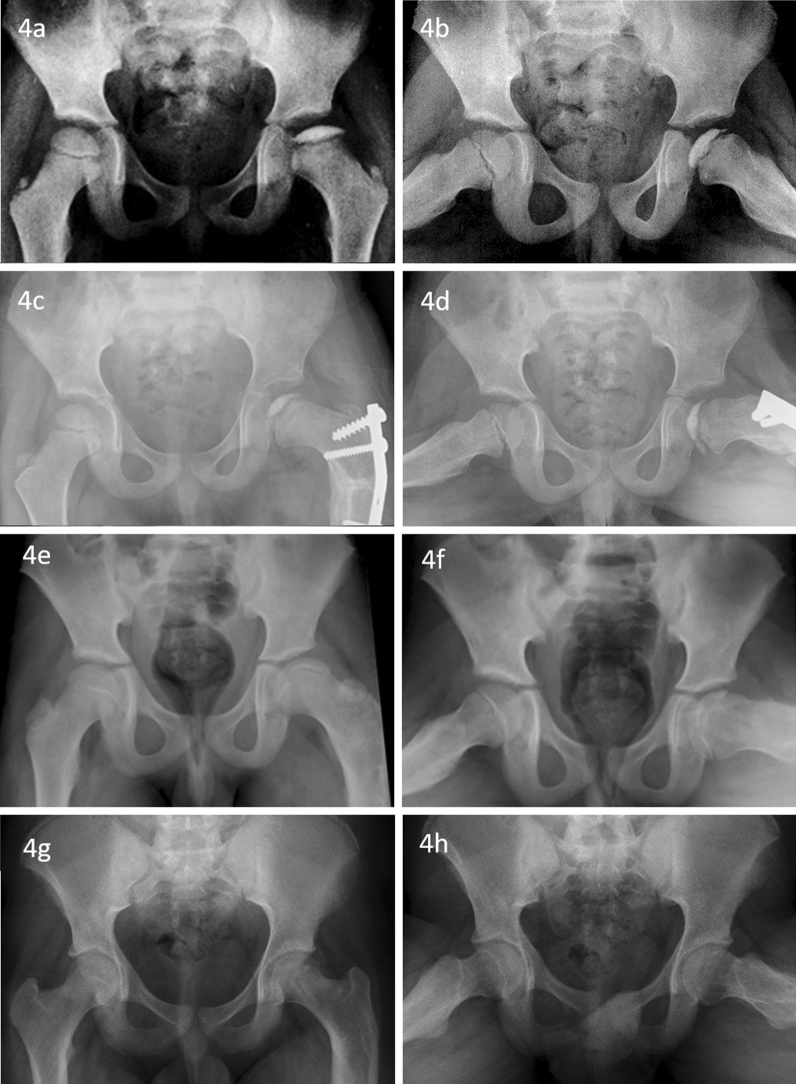
Anteroposterior (**a**) and frog-lateral (**b**) radiographs of a boy with the age of onset 5.5 years with left side Perthes disease (**a**, **b**). The Reimer’s index was 31.75 % and difference was 12.29 % (a). He underwent varus derotation osteotomy with trochanteric epiphysiodesis (**c**, **d**). The radiographs at healing (**e**, **f**) and skeletal maturity (**g**, **h**) showed good outcome

Children younger than 5 years with significant extrusion were managed with fixed abduction brace (35-degree hip abduction). They were treated with non-weight-bearing walking after 5 years of age till stage IIIb (Fig. 3). Older than 5 years’ age with significant extrusion at initial presentation or at subsequent follow-up till the stage of early fragmentation were treated with varus derotation osteotomy and trochanteric epiphysiodesis. Surgical containment was considered with 20 degrees pre-bend plate with sub-trochanteric open wedge osteotomy [13]. The surgical containment was done only in the early stage of the disease (Fig. 4). All children were kept non-weight bearing with axillary crutches till stage IIIb. Weight bearing was allowed at the stage IIIb of the disease.

These children were prospectively followed at 3 months interval during the active stage of the disease and thereafter annually till skeletal maturity. Skeletal maturity was defined as children with Oxford grading more than 17 [14]. All their serial radiographs were evaluated. The extent of head involvement was calculated during the stage of fragmentation by Catterall classification [2]. The extent of the head involvement was not considered for the decision making of the treatment.

We included children with early-stage presentation and followed till the skeletal maturity. The children with late presentation and not followed till skeletal maturity were excluded. Sixty-eight children formed the basis of the study. Out of 68 children (77 hip joints) with Perthes disease, nine children had bilateral involvement (all 18 hips got affected before the age of 7 years).

Clinical outcome at skeletal maturity was assessed by the presence of pain, limp, range of motion of the hip. The shape of the femoral head at skeletal maturity was assessed by Stulberg classification [15] and sphericity deviation score [16].

### Statistical analysis

Statistical package for social sciences software for Windows SPSS version 20.0 was used for statistical analysis. The independent “*t*” test and Chi-square test were used to compare the difference between the two groups.

## Results

The mean age at the onset was 5.71 ± 0.87 years (3–7 years) in 68 children. The frequency of significant extrusion was 57% in the study. The mean age of the children in both groups was comparable. Boys and girls were equally distributed in both groups. The mean age for the final follow-up was comparable, though the patients in the non-operative group had longer follow-up (Table 1).

**Table 1:** Demographic data of 68 children (77 hips) with Perthes disease onset before 7-year age

	Total	Significant extrusion* (*n* = 44)	No significant extrusion (*n* = 33)	*p*	Operative group (*n* = 38 hips)	Non-operative group (*n* = 39 hips)	*P*
Age of onset (years) (Mean ± SD)	5.71 ± 0.87	5.82 ± 0.91	5.57 ± 0.82	0.218	6.07 ± 0.67	5.37 ± 0.92	< 0.001
*The age of onset*							
< 5 years	12	7	5	0.357	1	11	< 0.05
5–6 years	26	12	14		12	14	
6–7 years	39	25	14		25	14	
Age at follow-up (years) (Mean ± SD)	15.28 ± 1.95	15.21 ± 2.23	15.38 ± 1.5	0.699	15.16 ± 2.16	15.40 ± 1.73	0.593
Duration of follow-up (years) (Mean ± SD)	9.32 ± 1.85	8.93 ± 1.89	9.84 ± 1.68	< 0.05	8.77 ± 1.93	9.87 ± 1.61	< 0.05
*Gender*							
Boys	45 (53 hips)	32	21	0.394	26	27	0.939
Girls	23 (24 hips)	12	12		12	12	
*Catterall*							
Group III	43	23	20	0.218	19	24	0.146
Group IV	31	21	10		19	12	

^*^Significant extrusion–migration difference more than 12% in unilateral involvement and Reimer’s index more than 15% in bilateral Perthes

**Fig. 5:**
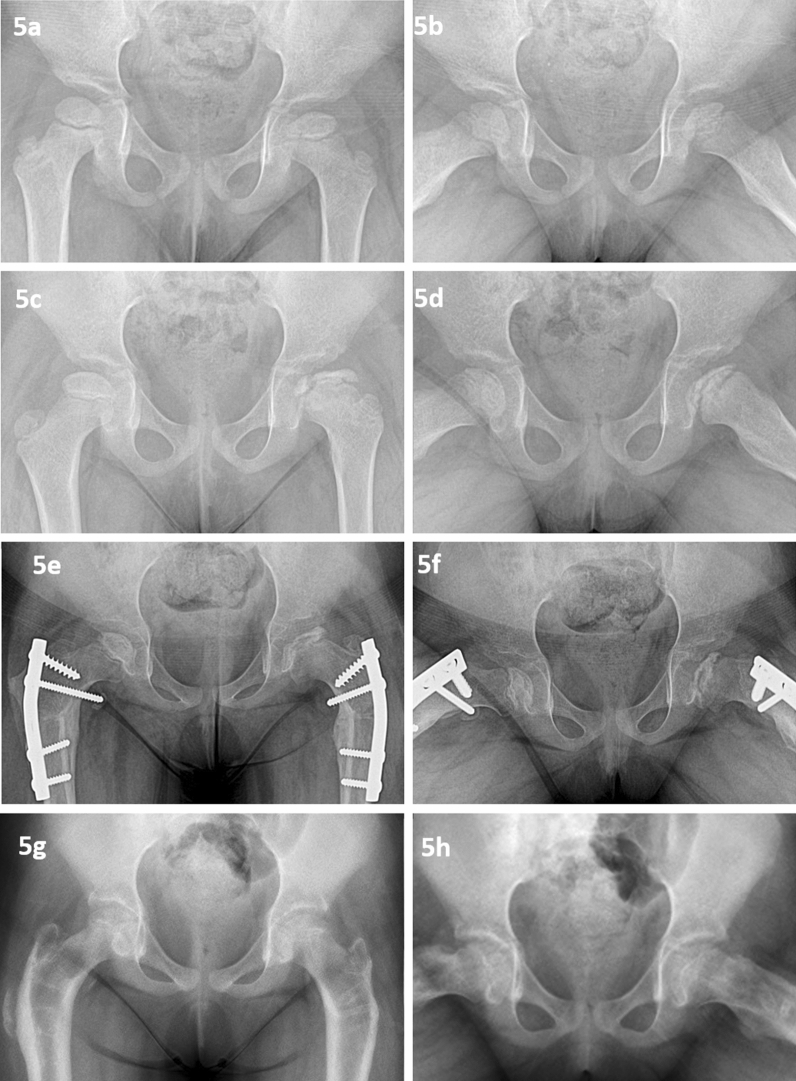
A girl presented with left hip Perthes with onset at 4.8 years and 11.44% extrusion (**a**, **b**). She was managed with abduction brace. Thirteen months later, right hip developed Perthes and extrusion in left hip increased to 36.10 % (**c**, **d**). Both hips required Varus derotation osteotomy and trochanteric epiphyseodesis (**e**, **f**). Both hips healed to spherical congruent hips (**g**, **h**)

Eleven children (12 hip joints) had less than 5 years of age at onset. Five children had a bilateral involvement. Seven out of twelve femur heads had a significant extrusion. Significant extrusion was noted in two hips with unilateral involvement and five hips with bilateral involvement. All these children were treated with a fixed abduction brace. Despite the abduction brace, extrusion increased in 13 months’ follow-up, and the second hip also developed Perthes in a girl. This girl underwent bilateral femur head containment surgery (Fig. 5).

**Fig. 6:**
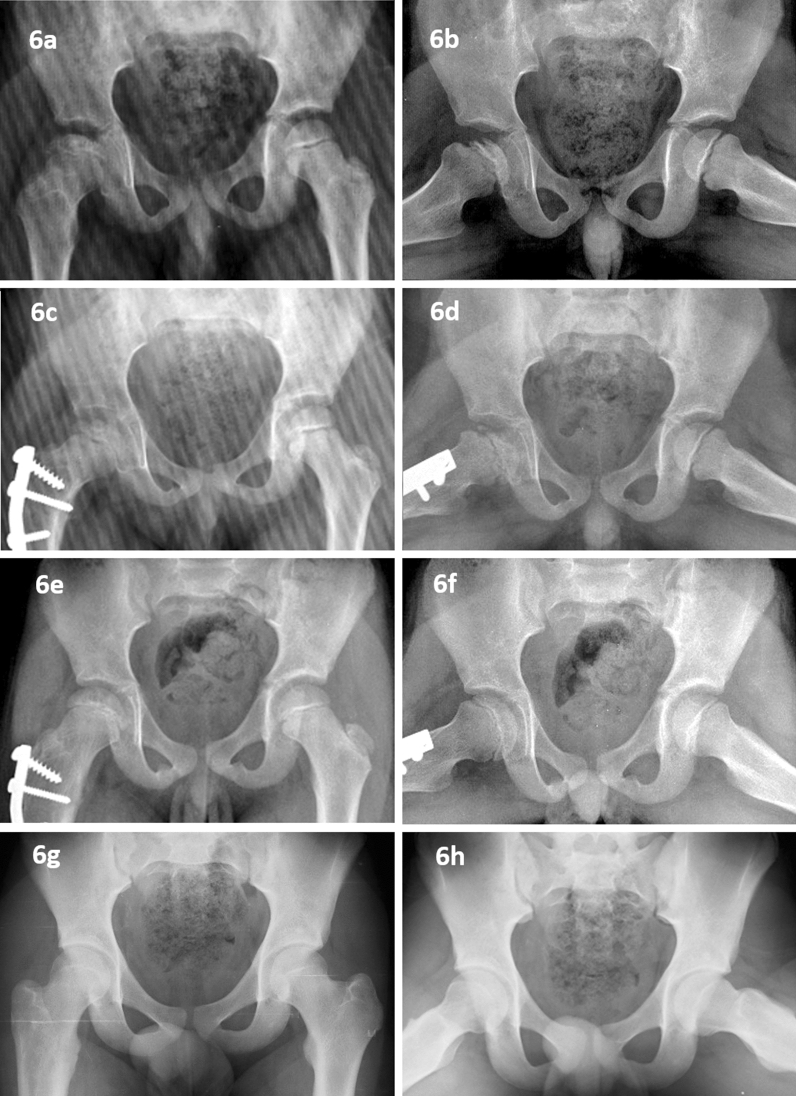
A 6.5-year boy presented with right hip Perthes disease in early stage with a significant extrusion (**a**, **b**). He underwent varus derotation osteotomy and trochanteric epiphysiodesis of right femur with 20-degree pre-bend plate (**c**, **d**). At skeletal maturity, good outcome (Stulberg Class II) was noted (**e**–**h**)

Fifty-seven children (65 hip joints) developed Perthes between 5 and 7 years of age. Thirty-seven hips had a significant extrusion. All these hips with a significant extrusion were treated with varus derotation osteotomy and trochanteric epiphysiodesis (Fig. 6). Ninety-six percent of children had more than half involvement during the stage of fragmentation; three hips were not classified due to bypass of the stage of fragmentation. There was no significant difference in the distribution of Catterall IV in both groups. The extent of involvement in both groups was comparable.

An excellent clinical outcome was noted at the final follow-up. No patients had pain, or restriction of range of motion. A mild limp was noted in 4 children. There was no significant difference in clinical outcomes between both groups. Radiographs at skeletal maturity showed good outcomes in 88.3% hips, and mean SDS was 8.6. All hip joints were congruent. Sixty-eight hips were spherical, whereas nine hips were oval. None of the femur head was flattened or incongruent, indicating an overall good to a fair outcome (Table 2). Comparable Stulberg groups and SDS between the two groups were noted (Figs. 2, 3, 4, 5, and 6). No variable, including the age of onset, gender, the extent of involvement, type of treatment, and significant extrusion at the beginning, significantly affects the radiological outcome at skeletal maturity (Table 2).

**Table 2:** Radiological outcome of Perthes children with the age of onset less than 7 years

	Stulberg			*P*	SDS (Mean ± SD)	*P*
	I	II	III			
All patients (*n* = 77 hip joints)	33	35	9	–	8.64 ± 10.82	–
*Gender*						
Boys (*n* = 53)	23	26	4	0.224	7.62 ± 7.99	0.332
Girls (*n* = 24)	10	9	5		10.89 ± 15.32	
Age at onset Years (Mean ± SD)	5.56 ± 0.87	5.75 ± 0.90	6.17 ± 0.65	0.172	0.079*	0.493
*Catterall*						
Group III (n = 43)	23	16	4	0.111	7.39 ± 7.99	0.501
Group IV (*n* = 31)	9	18	4		8.74 ± 9.09	
*Treatment*						
Non-operative (*n* = 39)	22	14	3	0.055	9.36 ± 12.20	0.556
Operative (*n* = 38)	11	21	6		7.90 ± 9.29	
*Extrusion*						
Significant extrusion (*n* = 44)	15	23	6	0.199	7.15 ± 8.85	0.165
No significant extrusion (*n* = 33)	18	12	3		10.62 ± 12.87	

^*^Pearson correlation

## Discussion

The natural history of early-onset and late-onset of the disease is different. The natural history of Perthes disease in the younger population is favorable. However, no consistent excellent results were noted in the literature. Poor results were noted in one-fourth of the population in children younger than 6 years, with groups III and IV Catterall [6, 17].

The age of onset of disease, the extent of involvement, lateral pillar, and extrusion of the femoral head play a significant role in the outcome of the disease [18–20]. Among these variables, the extrusion of the femoral head is only surgeon-dependent variable [19]. The exact frequency of extrusion of the femur head is poorly studied in the literature. The reversal of extrusion is associated with good outcome [21].

Varied literature showed different results in different populations. Canavase and Dimeglio, in their study, reported that in 166 hips with a mean age of onset 44 months, 128 (77%) hips had a good outcome [1]. Rosenfeld, Herring, and Chao analyzed 190 hips with the age of onset less than 6 years and concluded that the prognosis for a patient with the onset of Perthes disease before the age of 6 years was generally favorable, 80% having good results [7].

Gent et al., in their study of 69 hips with the age of onset less than 6 years, concluded that 45 hips (64%) had a good outcome with hips classed Stulberg I and II. The 14 hips (20%) classed Stulberg III, and the ten hips (14%) classed Stulberg IV had a poor outcome. They showed overall good results in children with the age of onset fewer than 6 years [9].

Fabry et al. studied 36 hips with Perthes disease. Of those with severe involvement, seven were in Catterall group III and 16 in group IV. At skeletal maturity, they found that 44% had good, 22% fair, and 33% poor results according to the Stulberg groups. Hence, they stated that a young age did not protect from the severe disease [6]. Snyder reported poor results in 43% of children with onset less than 5 years of age [8]. Nakamura, Kamegaya et al. assessed 114 hips in patients with the age of onset less than 6 years in which good outcome was observed in 72 hips (63%) with onset before 6 years of age [22]. The current study is compared with similar studies in the literature (Table 3).

**Table 3:** Comparison of results in younger children with Perthes disease

Author	Journal year	Total number of children	Total number of hips	Good results Stulberg I/II *N* %	Fair results Stulberg III *N* %	Poor results Stulberg IV/V *N* %
Nakamura et al. [22]	JPO 2015	100	114	72 63%	28 25%	14 12%
Canavese and Dimeglio [1]	JBJS Br 2008	146	166	78 67%	26 22%	12 11%
Gent et al. [9]	JCO 2007	67	69	45 65%	14 20%	10 15%
Rosenfeld et al. [7]	JBJS Am 2007	160	164	131 80%	14 8%	19 12%
Herring et al. [5]	JBJS Am 2004	176	180	109 61%	54 30%	17 9%
Fabry et al. [6]	JPO B 2003	30	36	16 45%	8 23%	12 33%
Present study	2021	68	77	68 88.3%	9 11.7%	0

There were 24 female hips out of 77 included in this study. This is an atypical sex ratio for Perthes' disease compared to almost all other studies. With more advanced skeletal maturation for the same chronological age, the high proportion of females could have worse results; we did not find the worst results in females (Fig. 6).

The effect of the extent of head involvement is well known. The poorer results were attributed to the severe involvement of the head. Most of the time, more severe cases were selected for surgery. As the extent of the involvement is categorized in the advanced stage of fragmentation, the extent of head involvement was not considered for decision making in this study. However, all children had more than 50% of head involvement. The frequency of Catterall groups was statistically comparable in both groups.

### The extrusion of the femoral head

The concept of “extrusion” is one of the signs of “head at risk” described by Catterall [2]. Green et al. first studied the effect of epiphyseal extrusion in the prognosis of Perthes disease [3]. They found more than 20% extrusion is associated with poor results. More than 20% extrusion in even Catterall group II had 40% good results. Only 8% of hips showed good results with 20% or more extrusion in Catterall group III/IV. The poor results were associated with more than 20% extrusion of the epiphysis. However, both studies did not describe the applicability with the stage of the disease. Coxa magna is associated with poor outcome. The coxa manga can present like the extrusion of the femur head in the late stage of the disease. Epiphyseal extrusion happens during the stage of avascular necrosis or the stage of fragmentation. The intervention in the early stage would prevent irreversible deformation of the femoral head. The intervention after irreversible changes would be too late for the prevention of deformation.

No study in the literature objectively quantifies the percentage difference to define the surgical containment. Eklöf et al. evaluated normal radiographs in all pediatric age groups [12]. They showed a difference of 12 or more percentage compared to the normal side was regarded as abnormal/subluxation. Hence, a difference of 12% or more migration was chosen as significant extrusion and the segregating factor for surgical indication.

The age of onset of disease, the extent of involvement, lateral pillar, and extrusion of the femoral head play a significant role in the outcome of the disease [17]. In this population, the younger age of onset is favorable. The extent of involvement and the lateral pillar are only classified in the advanced stage of fragmentation. The natural history of the disease cannot be altered by all non-dependent variables. Hence, the extrusion of the femoral head is only the surgeon-dependent variable. This study shows the usefulness of the surgeon-dependent factor “extrusion” in the early stage of the disease in this younger population. Non-weight-bearing crutch walking with surgical containment is useful in getting good results [22]. The good results in the study might be due to the younger onset of the disease, aggressive and early management of extrusion of the head, and prolonged non-weight-bearing crutch walking. Further studies are required to identify the effect of each independent variable on the prognosis of the disease.

### The effect of non-weight bearing

There is a lot of controversy regarding the impact of non-weight bearing associated with containment or without containment. Earlier studies showed no difference associated with or without non-weight bearing. However, a recent study on immature pigs showed non-weight bearing decreased deformity of the femoral head and increased revascularization rate [23]. The study simulates non-weight-bearing crutch walking than absolute bed rest. However, the local non-weight bearing must be prolonged until the advanced stage of revascularization to get the benefit of non-weight bearing. Few clinical studies with prolonged non-weight bearing showed good results of hip and mechanical alignment of the knee [24, 25].

In this study, 88.3% of good results (Stulberg I or II) were achieved in children younger than 7 years. The frequency of more extrusion was noted higher in older children than younger children. The incidence of more extrusion was observed higher in Catterall group IV.

The possibility of poor outcome in the Perthes disease might be related to extrusion of the epiphysis in localized manifestation of generalized disease. These children do no manifest the whole syndromic criteria of the disease. These children might be the carrier to the connective tissue disorder due to defective extracellular matrix as seen with COMP, COL2A1, COL9A1, COL9A2, etc. genetic mutation [26–29]. Whole exome sequencing with detailed genome analysis in such children might help to differentiate Perthes disease with localized manifestation of skeletal dysplasia [30].

### The uniqueness of the study

The present study reports excellent results in three forth of children population. Poor results (Stulberg IV and V) are not observed in the current study. The extent of involvement of the femur head is similar to other studies. The reason for the study's excellent and fair results is the aggressive treatment of the extrusion of disease in the early stage of the disease with prolonged non-weight bearing until stage IIIb. The extent of involvement and severity of collapse lead to poor results in Perthes disease irrespective of the age of onset of the disease. If we can avoid the severity of collapse and maintain the head's containment, it would be associated with good results. Hence, the poor results, like Stulberg IV/V, were not observed in this study.

### Limitation of the study

The children with significant extrusion were treated with containment in this study. Ideally, natural history can be studied without any intervention. However, due to previous experience, the intervention was justified to prevent poor outcomes. Further studies are required to detect the exact amount of extrusion or % difference between both sides to treat the children optimally.

## Conclusion

The outcome of the children who had the age of onset of the disease less than 7 years was good with early and aggressive management of the extrusion. The reversal of extrusion is associated with a similar result of non-operative children in this age group.
